# Influence of supplemental vitamin D on intensity of benign paroxysmal positional vertigo: A longitudinal clinical study

**Published:** 2016

**Authors:** Mahboobeh Sheikhzadeh, Yones Lotfi, Abdollah Mousavi, Behzad Heidari, Mohsen Monadi, Enayatollah Bakhshi

**Affiliations:** 1 Department of Audiology, University of Social Welfare and Rehabilitation Sciences, Tehran, Iran.; 2 University of Social Welfare and Rehabilitation Sciences, Tehran, Iran.; 3 Tehran University of Medical Sciences, Tehran, Iran.; 4 Mobility Impairment Research Center, Babol University of Medical Sciences, Babol, Iran.; 5 Department of Audiology, Babol University of Medical Sciences, Babol, Iran.; 6 Department of Statistics, University of Social Welfare and Rehabilitation Sciences, Tehran, Iran.

**Keywords:** Benign paroxysmal positional vertigo, Intensity, Vitamin D, Rehabilitation Therapy.

## Abstract

**Background:**

Benign paroxysmal positional vertigo (BPPV) is linked to vitamin D deficiency. This clinical trial aimed to determine the influence of vitamin D supplementation on intensity of BPPV.

**Methods:**

The study population was selected consecutively and the diagnosis of BPPV was made by history and clinical examination and exclusion of other conditions. Intensity of BPVV was assessed based on VAS score **(**0-10**)**. Serum 25-hydroxyvitamin D (25-OHD) was measured using ELISA method and levels < 20 ng/ml was considered a deficiency. All patients received rehabilitation treatment using Epley's maneuver one time per week for one month. Serum 25-OHD deficient patients were classified as treated and non-treated groups (rehabilitation with or without 50.000 IU cholecalciferol weekly for two months).The results of treatment were compared with vitamin D sufficient group as control. All patients were followed-up for 6 months.

**Results:**

After two months of treatment, in both vitamin D treated and non-treated groups the intensity of BPPV decreased significantly as compared with control (P=0.001 for both groups) but at endpoint, the intensity of BPPV aggravated and regressed to the baseline value in vitamin D deficient non-treated group (P=0.001) whereas, in vitamin D treated group, improvement of BPPV remained stable and unchanged over the study period.

**Conclusion:**

This study indicates that correction of vitamin D deficiency in BPPV provides additional benefit to rehabilitation therapy (Epley maneuver) regarding duration of improvement. These findings suggest serum 25-OHD measurement in recurrent BPPV.

Vitamin D deficiency is an important health problem over the world as well as in Iran (1, 2). Vitamin D deficiency is linked to development, progression or severity of several skeletal and non-skeletal conditions (3-6). Normalization of vitamin D deficiency with vitamin D supplement prevents disease progression or results in clinical improvement in many clinical conditions (4, 5, 7, 8). Benign paroxysmal positional vertigo (BPPV) is a relatively common condition affecting 2.4% of the general population (9). Frequent recurrent attacks of vertigo particularly in the elderly people affect the quality of life and results in impaired daily activities with consequent disability particularly in elderly people (10). In older subjects, vestibular dysfunction and dizziness are common causes of fall (11).

Results of a number of clinical trials demonstrated beneficial effect of vitamin D treatment on muscle strength of the lower limbs, body sway and physical performance in subjects older than 50 years (12). Similar observations regarding the beneficial effect of vitamin D therapy on muscle strength have been observed in a systematic review of 13 studies (13). Improvement of postural balance is an important component of treatment in older patients with dizziness and imbalance (11). Restoration of serum vitamin D improves muscle strength in lower limbs and is expected to improve balance and fall (7, 12).

In the geographic region of this study, vitamin D deficiency is common in the general population (1,2)and coexistence of BPPV with vitamin D deficiency is expected to be prevalent .Our observations indicate more severe BPPV in vitamin D deficient subjects. Nevertheless, data regarding vitamin D deficiency and vertigo are scarce and this context has not been adequately investigated. Currently, treatment of BPPV is based on rehabilitation therapy using Epley’s maneuver which has been shown to be safe and effective with recurrence rates of 12-36% across various studies (4, 5, 14-16). We proposed that raising serum vitamin D to sufficient levels may confer additional benefit to rehabilitation therapy, through strengthening of the lower limb muscles and improvement in neuromuscular function. 

For these reasons, the present study was conducted to examine the relationship between vitamin D deficiency and severity of BPPV and to determine the outcome of patients after correction of vitamin D deficiency

## Methods

The study population was selected consecutively among the patients presented to Ayatollah Rouhani Hospital ENT, neurology and audiology clinics over a one-year-period from April 2014 to April 2015.

All patients had history of at least two or more attacks of BPPV over 6 months prior to inclusion. Exclusion criteria included, patients with history of head trauma, surgery or infectious diseases of the ear, maxillary sinuses and patients with chronic renal, pulmonary, hematologic, gastrointestinal, cardiovascular diseases, taking supplementary calcium and vitamin D or taking medications which alter vitamin D metabolism. Data were collected through an interview, clinical examination, and laboratory tests. Intensity of BPPV was assessed by the patients and expressed as VAS score (0-10), which 0 indicated no vertigo and 10 indicated severe attacks of vertigo.

The sample size was estimated to detect 1 score difference in intensity of BPPV between patients with and without serum 25-OHD deficiency. Based on a standard deviation of 1.2, each group needed 23 patients for the detection of such difference with power of 80%, and 95% confidence interval. Diagnosis of BPPV was confirmed according to patient’s history and clinical examination after exclusion of other disease conditions. Serum 25-hydroxyvitamin D (25-OHD) was measured using ELISA method and concentrations less than 20ng/ml was considered deficiency (17)

All patients received rehabilitation treatment using Epley's method one time per week for one month. Patients with serum 25-OHD deficiency were classified as treatment group (Epley maneuver + supplemental vitamin D) or control group (Epley maneuver therapy alone). Vitamin D was administered at 50.000 IU weekly for two months and then 50.000 IU monthly over the 6-month study period. In statistical analysis, the distribution of all variables was determined using Kolmogorov-Smirnov test. The association between vitamin D deficiency and severity of BPPV was determined by comparing of BPPV patients with and without vitamin D deficiency. The influence of vitamin D on BPPV was determined by comparing patients with and without vitamin D supplementation at the end of the study period. Comparisons between groups for variables with and without normal distributions were performed using student t-test or Mann-Whitney U test and Kruskal-Wallis test, respectively. SPSS software Version 18 was applied for analysis.

## Results

Eighty-one patients (females 55.6%) entered to the study. Vitamin D deficient patients were classified as treated group (n=27) taking rehabilitation therapy and supplemental vitamin D and non-treated group (n=27) taking rehabilitation therapy alone. Patients with sufficient serum 25-OHD (n=27) were considered as the control group. 

At baseline the intensity of BPPV in patients with and without serum 25-OHD deficiency was comparable (table 1) without statistical difference (P=0.22). Similarly, at baseline, the intensity of BPPV between vitamin D deficient groups (treated and non-treated groups) did not differ with the control group (P=0.15, and 0.39), table 1. 

**Table 1:** Comparison of baseline vitamin D deficient and sufficient ^¥^ patients with intensity of benign paroxysmal positional vertigo (BPPV) according to intensity of BPVV

**Groups**	**25-OHD ** ** ng/ml**	**BPPV ** β **VAS (0-10)**
Vit D deficient (n=54	11 (7-19)	7 (5-9)
Vit D sufficient (n=27)	32 (25-55)	7 (5-9)
P values α	0.001	0.22

¥ Serum 25-OHD < 20 mg/ml was considered as deficient and levels >30 ng/ml as sufficient

β Assess by the patients and scored by VAS(0-10)

α Assessed by the patients prior to inclusion as well as over the study period

α Compared with Mann-Whitney U test

After treatment with vitamin D, serum 25-OHD in the vitamin D treated group increased significantly from 11.4±1.9 ng/ml at baseline to 34.2+3.3 at month 2 (p=0.001) whereas, in non-treated and control groups serum 25-OHD remained unchanged (table 2). 

After two months of treatment the intensity of BPPV decreased significantly in all study groups as compared with baseline (table 3). 

Over the study period, the intensity of BPPV aggravated and regressed to baseline value in vitamin D non-treated group (P=0.001) whereas, in the treated group the improvement of BPPV remained stable and unchanged over the 6–month study period as compared with the control group (p=0.001) (table 3).

**Table 2:** Serum 25-hydroxyvitamin D status in patients with benign paroxysmal positional vertigo (BPPV) under rehabilitation therapy with and without supplemental vitamin D supplementation over 6 months follow-up duration

**BBPV groups**	**Types of treatment**	**Baseline** **Mean** **+** **SD**		**Month 2** **Mean** **+** **SD**		**Month 6** **Mean** **+** **SD**	
Vitamin D deficient¥ (treatment group) (n=27)	Rehabilitationµ+vitamin D&	11.4+1.9	0.001	34.2+3.3	0.88	35.5+2.9	0.35
Vitamin D deficient (non-treatment group) (n=27)	Rehabilitation µ alone	10.7+2.3	0.001	10.6+3	0.001	11.1+2.3	0.001
Control (n=27)	Rehabilitation µ alone	33.8+6.6	-	34.4+6	-	36.2+5.4	-

pβ Compared with baseline

P* Comparison with the control group by Student t test

¥ Serum 25-OHD < 20 mg/ml

µ Epley therapy

& Cholecalciferol 50.000 IU weekly for two months

**Table 3:** Influence of supplemental vitamin D on intensity of benign paroxysmal positional vertigo (BPPV) under rehabilitation therapy over 6 months follow-up duration

**BBPV groups**	**Types of treatment**	**Baseline** **Mean** **+** **SD** **Median(range)**		**Month 2** **Mean** **+** **SD** **Median(range)**	**pβ**	**Month 6** **Mean** **+** **SD** **Median(range)**	
Vitamin D deficient ¥ (treatment group) (n=27)	Rehabilitation µ + vitamin D	7.+1.3 7(5-9)	0.15	0.44+0.80 0(0-3)	0.001	0.22+0.42 0(0-1)	0.001
Vitamin D deficient(non-treatment group) (n=27)	Rehabilitation µ alone	7.22+1.01 7(5-9)	0.39	0.22+0.42 0(0-1)	0.001	6.9+0.94 7(5-9)	0.001
Control (n=27)	Rehabilitation µ alone	6.8+1.01 7(5-9)	-	0.26+0.52 0(0-2)		0.3+0.82 0(0-3)	-

p^β^ Compared with baseline

P* Comparison with the control group by Student t test

¥ Serum 25-OHD < 20 mg/ml

µ Epley therapy

& Cholecalciferol 50.000 IU weekly for two months

## Discussion

The results of this study demonstrated a significant decrease in the intensity of BPPV two months after treatment in all groups irrespective to serum 25-OHD status. Thereafter, improvement persisted in vitamin D sufficient and vitamin treated groups but regressed to baseline value in vitamin D deficient group. Based on the results of this study, Espley’s therapy is effective in the treatment of BPPV for a short time period but persistent of improvement requires normalization of serum vitamin D in those who have vitamin D deficiency. Therefore, rehabilitation therapy in vitamin D deficient patients exerts a short-term beneficial effect but correction of deficiency with supplemental vitamin D confers additional benefits for loger period. Several observations support the results of this study. Talal et al. in a follow-up study of vitamin D deficient patients demonstrated that raising serum 25-OHD > 10 ng/ml increase in serum 25-OHD by treatment significantly decreased the recurrences as well as the number of BPPV attacks as compared with those who had less than 10 ng/ml increment (18). Jeong et al. showed that in patients with serum vitamin D between 10-20 ng/ml, the risk of BPPV increases 3.8 times, whereas, in patients with serum vitamin D, less than 10 ng/ ml, the risk increases by odds of 23 (19).

The beneficial effect of vitamin D therapy on severity of BPPV may be attributed to direct effect of vitamin D on vestibular system or indirect effect of vitamin D, on muscle strength, fall, balance and musculoskeletal system (20, 9, 10, 21). Serum concentrations of 25-OHD > 30 ng/mL were consistently associated with improvement in muscle strength and balance. This may explain the mechanistic basis of fall prevention with higher doses of vitamin D. Optimal fall prevention has been found in studies that achieved mean serum 25-OHD up to 75 - 100 nmol/L, whereas serum 25-OHD < 60 nmol/L did not reduce falls (22). Muscle weakness and balance disorder have a role in completing vertiginous attacks.Therefore strengthening effect of vitamin D on muscle may exert some beneficial effects on vertiginous attacks (23, 24). 

In mice, vitamin D receptor deficiency is associated with balance impairment. Similarly, vitamin D deficiency may also predispose humans to impaired balance/posture controls (9). Vitamin D is necessary for calcium and bone hemostasis so there is a correlation between biomarkers of bone turn over and BPPV (25). Low level of vitamin D is associated with both low bone mass and recurrence or development of BPPV (26). In a review of 101 cases, BPPV was more frequent in women at postmenopausal age (9). A systematic review of seven studies demonstrated a positive correlation between low bone mass and BPPVe especially in older women (27). Low bone mass is prevalent during postmenopausal women due to hormonal and reproductive factors (28, 29). The clinical significance of awareness to serum 25-OHD in patients with BPPV is not limited to prevention of BPPV recurrent attacks, but several extraskeletal consequence of vitamin D may improve by raising serum 25-OHD to sufficient levels (1, 2, 5-8, 29-32). This study has several limitations in respect to many comorbidities such as obesity, hypertension, metabolic syndrome and diabetes which are common in the geographic region of this study even in young individuals (33, 34, 35). These conditions may be associated with several nonspecific symptoms mimicking vertigo or differently affect the course of BPPV. However, in one study prevalence of comorbidities in BPPV did not differ with the general population (36). Nonetheless, in another study prevalence of metabolic syndrome was more common among male patients with vertigo as compared with general adult male (37). Nevertheless, the results of this study are less subject to be affected because, distribution of comorbidities is expected to be similar across comparison groups. In conclusion the results of this study indicated that rehabilitation therapy of BPPV in vitamin D deficient state exerts a temporary beneficial effect on severity of BPPV and correction of vitamin D deficiency with supplemental vitamin D reduces recurrent attacks and provide improvement for longer duration. This context requires further longitudinal studies.

## Notes

**Funding: **This article is result of Dr Sheikhzadeh Thesis.
